# Elevated Circulating Endocan Levels Are Associated with Increased Levels of Endothelial and Inflammation Factors in Postprandial Lipemia

**DOI:** 10.3390/jcm12041267

**Published:** 2023-02-06

**Authors:** Serap Ozer Yaman, Fulya Balaban Yucesan, Cihan Orem, Birgul Vanizor Kural, Asım Orem

**Affiliations:** 1 Department of Medical Biochemistry, Faculty of Medicine, Karadeniz Technical University, Trabzon 61080, Turkey; 2 Department of Cardiology, Faculty of Medicine, Karadeniz Technical University, Trabzon 61080, Turkey

**Keywords:** endocan, endothelial dysfunction, inflammation, postprandial lipemia

## Abstract

Background: Postprandial lipemia (PPL) causes endothelial dysfunction by causing endothelial damage to lipoproteins that remain rich in triglycerides. Endocan is a proteoglycan with increased tissue expression, endothelial activation, and neovascularization. The aim of the study was to examine circulating endocan levels in PPL subjects by considering the degree of PPL response according to a high-fat test meal. The other aim was to determine the association between endocan levels and endothelial and inflammatory factors. Method: Fifty-four hyperlipidemic subjects and 28 normolipidemic subjects consumed the high-fat meal. Endocan, sICAM-1, sVCAM-1, and VEGFA as endothelial factors and IL-6 and LFA-1α as inflammatory factors were evaluated. Results: Fasting serum endocan, VEGFA, sICAM-1, sVCAM-1 IL-6, and LFA-1α levels were increased in the PPL group compared to the control group. The PPL group was divided into tertiles based on mean AUC levels. Endocan levels in tertile 3 were at the highest and were increased significantly compared to tertiles 1 and 2. AUC and endocan levels were positively correlated with other endothelial and inflammation factors. ROC analysis showed endocan levels to be one of the highest values. Conclusions: Circulating endocan is seen at significantly higher levels and independently associated with endothelial and inflammatory factors in postprandial lipemia and dyslipidemia.

## 1. Introduction

Postprandial lipemia (PPL) or postprandial hypertriglyceridemia exhibits the fluctuation and elevation of blood lipid levels between the postprandial period and the pre-meal level. It is defined by increasing levels of triglyceride (TG)-rich lipoproteins such as remnant-like lipoprotein particles (RLPs) [1,2]. Increasing RLPs in the postprandial period make a significant contribution to the development of atherosclerotic cardiovascular disease (ASCVD) [3,4], closely related to endothelial dysfunction, decreased HDL levels, increased oxidative stress, and inflammation [5,6,7]. Endothelial dysfunction plays a significant part in the pathophysiology of cardiovascular diseases (CVDs). It is thought to be the most important link between postprandial status and atherosclerosis [7,8]. In addition, the produced postprandial remnants induce the expression of leukocyte adhesion molecules in the endothelial cells, increasing the entry of inflammatory cells into the lesion site. Activated leukocytes in PPL contribute to endothelial dysfunction, with an increase in proinflammatory cytokines and oxidative stress at this site [6,7,8,9]. 

Endothelial-cell-specific molecule-1 (ECSM-1 or endocan) is a recently identified proteoglycan released from the vascular endothelium and associated with increased tissue expression, endothelial activation (inflammation), and neovascularization (tumor progression) [10]. It has been shown that increased endocan level is associated with the development and progression of atherosclerosis and CVD [11,12,13]. It was recommended that endocan can be a sensitive molecule of endothelial activation due to the detection of low levels of endothelial cells under physiological conditions compared to their elevation during inflammation and angiogenesis [14,15]. Endothelial dysfunction is associated with increased release of many proinflammatory factors such as vascular cell adhesion molecule (VCAM-1), intercellular adhesion molecule (ICAM-1), P-Selectin, and E-Selectin [15,16,17]. Endocan stimulates vascular smooth muscle cell proliferation. Thus, it provides the formation of the neointima layer at the stage of atherogenesis, and migration formation is observed in this process. It is also said to be one of the modulatory factors in the migration and adhesion of leukocytes to the endothelium [17,18]. In the regulation of these events, endocan indirectly increases the release of vascular endothelial growth factor (VEGF) and induces an increase in vascular permeability. On the other hand, VEGF similarly triggers endocan secretion. Endocan both plays a role in the recruitment of circulating lymphocytes into the inflammatory area and acts on lymphocte-function-associated-antigen-1 (LFA-1)-based leukocyte adhesion and activation. These pathways are involved in early atherogenesis and show that the endocan is involved in the pathophysiological mechanisms that are effective in the onset of atherosclerosis [19,20].

In view of the current data, increased serum endocan levels were found to be correlated with coronary artery disease (CAD), hypertension, diabetes mellitus (DM), obesity, chronic kidney disease, and end-stage renal disease [19,20,21,22,23,24,25]. However, there was no previous report investigating serum endocan levels in PPL and its possible association with endothelial dysfunction and inflammation. The current study was therefore proposed to evaluate circulating fasting endocan levels in individuals with PPL by considering the degree of PPL response (area under the curve (AUC)) according to the response to a high-fat test meal. It also aimed to evaluate the relationship of endocan levels with endothelial factors (ICAM-1, VCAM-1, and VEGFA) and inflammatory parameters such as IL-6 and LFA-1.

## 2. Materials and Methods

### 2.1. Subjects and Study Groups

Individuals in the current study group were selected from the Cardiology Polyclinic of Karadeniz Technical University, Faculty of Medicine. These individuals were selected for being normolipidemic, with fasting TG levels <1.69 mmol/L (<150 mg/dL), and hyperlipidemic, with fasting TG levels >1.69 mmol/L (>150 mg/dL). The panel of experts described that a TG concentration of >2.5 mmol/L (220 mg/dL) at any time after high-fat meal administration is a high and undesirable postprandial TG response [26]. Accordingly, the volunteers in our study were divided into control and PPL groups according to their TG levels 4 h after consuming the high-fat meal as follows. 

Control group: PPL testing negative for TG level ≤ 2.5 mmol/L 4 h after the high-fat meal; 28 normolipidemic subjects (14 female, 14 male) were included. 

**Figure 1:**
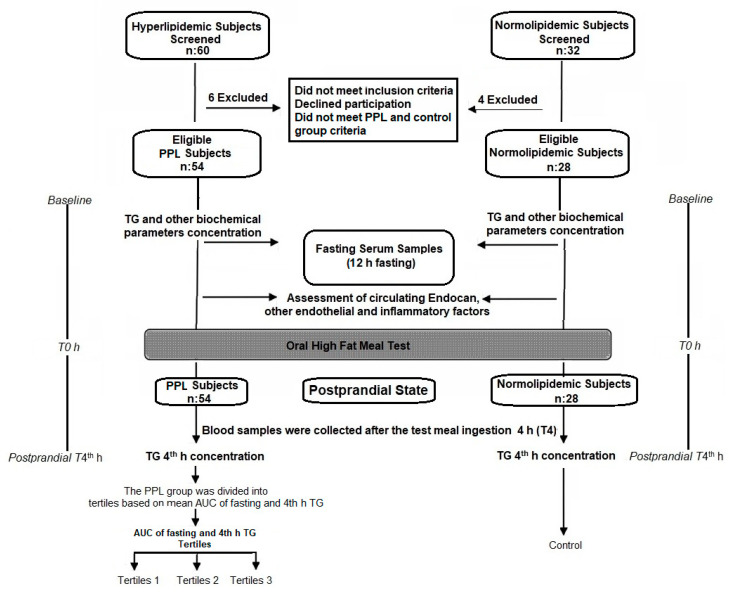
Flowchart showing control and PPL groups included in the study.

PPL group: PPL testing positive for TG level > 2.5 mmol/L 4 h after the high-fat meal; 54 hyperlipidemic subjects (26 female, 28 male) were included. The area under the curve (AUC) values of serum TG levels were calculated for 2 h fasting and 4 h after the high-fat test meal using the trapezoidal rule [2,27]. The tertile was divided according to AUC values to evaluate the degree of posprandial lipemia response in the PPL group. Figure 1 shows the flowchart of participants through the study.

Subjects were excluded if they were taking any medication other than dietary supplementation or if they were menopausal, on estrogen replacement therapy, pregnant, lactating, alcohol abusers, smoking, performing heavy exercise, or if they had a medical disease such as acute/chronic inflammatory diseases, kidney disease, hepatic failure, obesity, DM, lipid and lipoprotein metabolism disorders, or thyroid hormone disorders. 

Subjects’ height and body weight before the test meal were calculated. Body mass index (BMI) was calculated using the formula weight/height^2^ (kg/m^2^). Waist circumference was calculated at a level between the lower rib margin and the iliac crest using a plastic tape measure. Hip circumference was also calculated at a level between the iliac crest and thigh region. The waist circumference was divided by the hip circumference to calculate waist-to-hip ratio (WHR). The waist circumference was also divided by the height to obtain waist-to-height ratio (WHtR).

The study was approved by the ethics committee of the Karadeniz Technical University Faculty of Medicine Ethical Committee for Research on Humans (Submission Number 2021/401, dated 27 December 2021) and conducted in accordance with the guidelines in the Declaration of Helsinki. All patients voluntarily participated in the study and provided written informed consent.

### 2.2. Oral High-Fat Meal Test

On the test day, blood samples were collected at 8:30 am after a 12 h overnight fast. Each participant was given a test meal (high-fat meal) containing toast and liquid ‘ayran’ (200 mL). Participants consumed the high-fat meal within 20 min, as directed by the expert panel [26]. The contents of the test meal consisted of toasted bread, cheese, and butter. Firstly, this meal was heated in a toaster, and the cheese and butter were integrated with the toasted bread. In addition, to increase both the digestibility and tolerability of the toast, 200 mL ayran was given. Ayran drink is a traditional homogeneous liquid beverage made from yogurt and water. All test foods and beverages were determined to be commercial products with certain ingredients approved by the Ministry of Food, Agriculture and Livestock of the Republic of Turkey. The preparations of these foods and beverages were made by taking into account the content of the quantity calculation. Based on our previous studies [2,27], a standardized high-fat meal was prepared with a content of 22.7% carbohydrates, 68.2% fat, and 9.1% protein (75 g fat, 25 g carbohydrates, and 10 g protein), with a total energy of 815 kcal. Blood samples were drawn before the test meal ingestion (fasting—T0) and 4 h (T4) thereafter. All subjects’ test meal was well-tolerated and eaten by each. After the ingestion of the test meal, subjects were only allowed to drink water during the following 4 h. In the postprandial period, the participants continued their daily routines; heavy exercise was not allowed. A second blood sample was drawn from the participants 4 h later. The samples to be used for analyses were kept at −80 °C. As in our previous studies of postprandial lipemia, total TG concentrations (area under the curve—AUC) were computed using the trapezoidal rule to determine the magnitude of change during 12 h fasting and 4 h after the high-fat test meal. [2,27]. The PPL group was divided into three equal subgroups (tertiles) based on mean AUC levels.

### 2.3. Biochemical Parameters

Blood samples were drawn via a venipuncture without any anticoagulant tubes for serum and EDTA—anticoagulant tubes for plasma. All blood samples were centrifuged at 1800 g for 10 min. After separation of serum and plasma samples, they were frozen at −80 °C until analysis. Glucose, lipid (TC and TG), and lipoprotein (HDL-C and LDL-C) levels were determined using the AU5800 auto analyzer (Beckman Coulter, Shizuoka, Japan). Insulin level was measured using the IMMULITE 2000 XPi analyzer (Siemens, Munich, Germany). To evaluate insulin resistance, the homeostasis model assessment of insulin resistance (HOMA-IR) was used. The formula used for calculating the index was as follows: fasting serum insulin (μU/mL) × fasting plasma glucose (mmol/L)/22.5 [28]. Fasting remnant lipoprotein cholesterol (RLP-C) was calculated using the formula TC˗(HDL-C + LDL-C) [29], and the atherogenic index of plasma (AIP) was calculated as log (TG/HDL-C) [30].

### 2.4. Assessment of Endothelial Factors

Endocan, sICAM-1, sVCAM-1, and VEGFA were measured by a sandwich enzyme immunoassay ELISA kit (ELK Biotechnology, Cat No: ELK3625, Lot No:20324315751, Cat No: ELK9222, Lot No: 20324330706, Cat No: ELK1256, Lot No: 20324311250 and Cat No: ELK1129, Lot No: 20324311051, Wuhan, Hubei, China, respectively). The samples’ absorbance was measured at 450 nm using a spectrophotometer device with a microplate reader (VERSA max, Molecular Devices, San Jose, CA, USA). Intra-assay values of endothelial parameters for these tests were 6.27, 7.94, 7.10, and 6.61% CV, respectively. Endocan, sICAM-1, and VEGFA results were expressed as pg/mL, while the sVCAM-1 result was expressed as ng/mL.

### 2.5. Assessment of Inflammatory Factors 

IL-6 and LFA-1α were determined by sandwich ELISA kits (ELK Biotechnology, Cat No: ELK1156, Lot No: 20324311104, and Cat No: ELK1774, Lot No: 20324312256, Wuhan, Hubei, China, respectively). The sample absorbance was measured at 450 nm using a spectrophotometer device with a microplate reader (VERSA max, Molecular Devices, San Jose, CA, USA). Intra-assay values of inflammatory parameters for these tests were 5.44 and 6.52% CV, respectively. Results were expressed as pg/mL and ng/mL, respectively.

### 2.6. Statistical Analysis

All data were examined using IBM SPSS Statistics for Windows (version 23.0; IBM Corp., Armonk, NY, USA). The distribution of variables was assessed using the Shapiro–Wilk test. Normally distributed variables were expressed as mean ± standard deviation and non-normally distributed variables as median (interquartile range, 25–75% (IQR)). In the comparison of two independent variables, Student’s *t*-test was used for the parameters with normal distribution, while the Mann–Whitney U test was used for the evaluation of two independent variables with non-normal distribution. The “Kruskall-Wallis” test was used to evaluate more than two independent variables with non-normal distribution, and the “Mann-Whitney U” test was used for the post hoc variable. The PPL group was divided into three equal subgroups according to their AUC values. The mean AUC value was expressed as tertile 1 with the lowest value, tertile 2 with the intermediate value, and tertile 3 with the highest value. The comparison of these three groups was evaluated using the Kruskal–Wallis test. The receiver operating characteristic (ROC) curve analysis was evaluated using MedCalc Statistics Software version 19.1 (Medcalc software BVBA, Belgium). Spearman correlation analysis was performed to assess the relationships between endothelial and inflammatory factors with AUC and endocan in the PPL group. The sample size was measured using G*PowerSoftware 3.1 (Heinrich-Heine Universitat, Dusseldorf, Germany) to determine significant difference between the groups according to endocan levels with a moderate effect. There were 26 individuals in the control and PPL. The minimum sample number in each group was calculated by taking moderate effect size Cohen’s d = 0.8, alpha = 0.05, 1 − β = 0.80 e and sample size ratio = 1. Statistical significance was assessed at *p* < 0.05.

## 3. Results

The anthropometric characteristics, fasting lipid profile, and glucose and insulin levels of the present study group are given in Table 1. When the age parameter was analyzed between PPL and control groups, no significant difference was observed (*p* = 0.786). Fasting levels of TG and the 4 h TG (postprandial lipemia) were significantly higher in the PPL group compared with the control group (*p* = 0.0001, Table 1). Mean TC and LDL-C levels were higher, while the mean HDL-C level was lower in the PPL group than in the control group (*p* = 0.0001, 0.0001, 0.005, respectively, Table 1). The PPL group showed significantly higher fasting RLP-C and AIP levels compared to the control group (*p* = 0.002, 0.0001, respectively, Table 1).

Fasting serum endocan levels were increased in the PPL group compared to the control group (*p* = 0.0001). Significant differences were observed in other endothelial factors between the PPL and control groups (Table 2). Fasting serum VEGFA, sICAM-1, and sVCAM-1 levels in the PPL group were higher than in the control group (*p* = 0.0001). When examining inflammatory factors, the PPL group demonstrated significantly higher fasting serum IL-6 and LFA-1α levels compared to the control group (*p* = 0.0001).

The PPL group was divided into tertiles based on mean AUC levels. Endocan levels in tertile 3 showed the highest level and were increased significantly compared to tertiles 1 and 2 (*p* = 0.0001, Table 2). sICAM-1 and sVCAM-1 levels of tertile 3 increased significantly compared to tertiles 1 and 2, while VEGFA levels increased significantly only compared to tertile 1 (*p* = 0.0001, Table 2). Particularly noteworthy results were the levels of endothelial factors in tertile 3, which were found to be approximately 2-fold higher than tertile 1 in the PPL group (*p* = 0.0001, Table 2). When evaluated in terms of inflammatory factors, IL-6 and LFA-1α levels were observed to be significantly higher in tertile 3 than in tertile 1 (*p* = 0.001, Table 2). 

**Figure 2:**
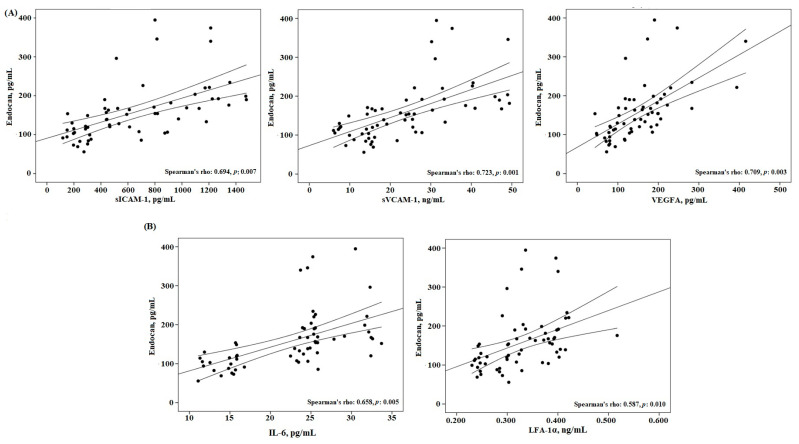
The correlation between (**A**) endocan and endothelial factors and (**B**) endocan and inflammatory factors in PPL group.

The Spearman correlation was performed to state the relations of AUC and endocan with endothelial, inflammatory, and atherogenic factors. As shown in Table 3, a positive correlation with AUC was determined with endothelial (endocan, sICAM-1, sVCAM-1, VEGFA) and inflammatory (IL-6, LFA-1α) factors (*p* = 0.003, 0.001, 0.001, 0.008, 0.006, 0.004, respectively). Endocan level was positively correlated with other endothelial factors (sICAM-1, sVCAM-1, VEGFA), especially inflammation factors (IL-6, LFA-1α, *p* = 0.007, 0.001, 0.003, 0.005, 0.010, respectively, Figure 2, Table 3). AUC and endocan levels were positively correlated with atherogenic factors (RLP-C and AIP, *p*= 0.037, 0.031, 0.001, 0.001, respectively, Table 3).

**Figure 3:**
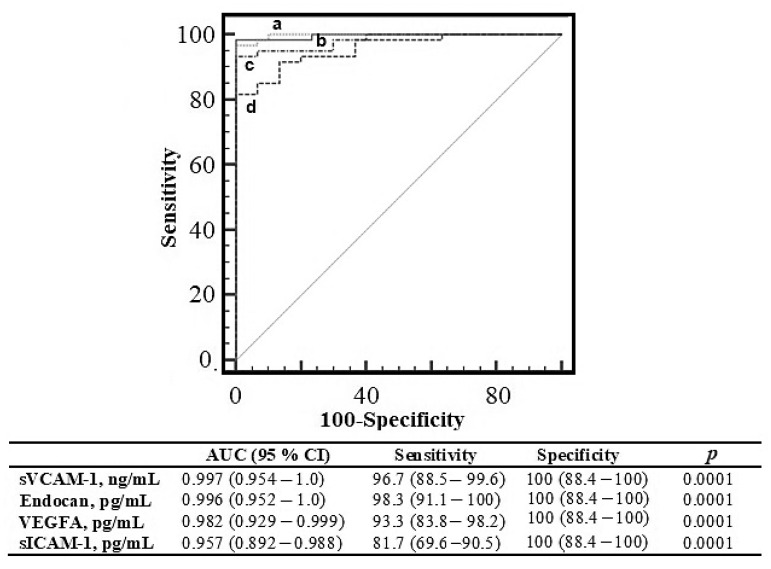
Receiver operating characteristic curve analysis of sVCAM-1 (a), endocan (b), VEGFA (c), and sICAM-1 (d).

The ROC curve result was obtained for the endocan and other endothelial factors in the PPL group and summarized in Figure 3. The AUC value of endocan levels was found to be the highest among other parameters with sVCAM-1 (AUC = 0.996, *p* = 0.0001).

## 4. Discussion

Postprandial TG concentration is a significant independent risk factor for ASCVDs. RLPs are increased significantly in the postprandial period and play a major role in the development of atherosclerosis [2,3]. PPL causes endothelial damage to remnant lipoproteins rich in TGs, resulting in the endothelial dysfunction encountered in the early development of atherosclerosis [3,6]. PPL is closely related to other diseases including endothelial dysfunction, oxidative stress, inflammation, and other mechanisms [8]. In order to understand the endothelial dysfunction involved in postprandial lipemia, the levels of serum endothelial derivate proteins and their association with inflammatory factors that may be involved in the pathophysiological changes observed in subjects with PPL were examined within the framework of the present study. In addition, the relationship of fasting endocan and other endothelial and inflammatory factors with the degree of PPL was evaluated in cases with impaired postprandial lipemia response.

In our study, it was observed that TC, LDL-C, and TG levels were higher and HDL-C levels were lower in the PPL group compared to the control group (Table 1). Moreover, postprandial 4 h TG levels were approximately 2-fold higher in PPL compared to the control groups (Table 1). TG levels after 4 h were 1.46 ± 0.414 and 2.94 ± 1.07 mmol/L in the control and PPL groups, respectively. The expert panel defines a high and undesirable postprandial TG response with a TG concentration >2.5 mmol/L (220 mg/dL) at any time after high-fat meal administration [26]. Accordingly, after consuming the high-fat meal, the subjects were selected as control or PPL and divided into groups. In addition, in our study on postprandial TG ranges in healthy subjects, 4 h TG levels were found to be 1.92 ± 1.11 mmol/L in healthy subjects [2]. Thus, it can be observed that in both control and PPL groups, 4 h TG levels are compatible with the literature. Elevated TG and decreased HDL-C levels were stated to be associated with endothelial dysfunction, increased oxidative stress, and atherogenic tendency [3,4].

The main purpose of this study was to examine circulating endocan levels in the PPL group. In this regard, circulating endocan levels were observed to be approximately 3-fold higher in the PPL group than in the control group (Table 2). Some novel studies have reported higher endocan levels in CAD, hypertension, DM, obesity, and chronic kidney disease compared to healthy groups [19,24]. In addition, He et al. demonstrated that serum endocan levels in large-artery atherosclerotic stroke patients are significantly increased [31]. Kose et al. confirmed that serum endocan levels were significantly increased in acute coronary syndrome [32]. Similarly, in addition, other studies stated that circulating endocan levels were significantly increased in the CAD compared, and this increase was consistently elevated [33,34]. In addition, in animal studies, high endocan levels were also emphasized in the early stages of atherosclerotic plaque development in mice lacking ApoE/LDL receptors. [35]. In the current study, circulating endocan levels were determined to be similar to the aforementioned studies, and these levels were associated with some degree of endothelial dysfunction and inflammation. 

To investigate the relationship of circulating endocan levels with the degree of PPL according to postprandial TG response, the PPL group was divided into tertiles according to AUC levels. For this reason, AUC levels were divided into subgroups (tertiles) to independently evaluate and understand the relationship between postprandial lipemia and endothelial dysfunction in the PPL group. One of the most important results in this study was showing that serum endocan levels gradually but significantly increase from the lower to upper tertiles (Table 2). In support of this finding, serum endocan levels were significantly positively correlated with AUC levels in the PPL group (Table 3). It was therefore suggested that endocan level may be closely related to PPL and its degree. In the light of these findings, there are many mechanisms that can clarify the present of increased serum endocan levels in PPL and its association with the development of increased atherosclerosis in PPL. Endocan may perform a major role in the development of endothelial cell dysfunction in PPL by promoting inflammation and cell adhesion as seen in many pathological conditions [18,19].

Adhesion of leukocytes via cell adhesion molecules and their complementary ligands includes inflammation promoted by TNF-α and IL-6 metabolism, which induces the expression of VCAM-1 and ICAM-1 in the endothelium [18]. As prior studies have indicated, endocan regulates the adhesion molecules’ expression and integrin-based cell adhesion [18,19]. In particular, it has been reported that by increasing the expression of ICAM-1, VCAM-1, VEGFA, and E selectin, this enables monocytes to adhere to endothelial cells, increase TNF-a release, and increase vascular proliferation and permeability and leukocyte migration. These studies demonstrated that endocan is involved in endothelial cell function. It affects and accelerates these processes, especially by promoting vascular and inflammatory mechanisms, as well as oxidative stress [33,36,37]. Our findings showed that sICAM-1, sVCAM-1, and VEGFA levels were increased in the PPL group and even increased gradually from the lower tertile to the upper tertile in the PPL group (Table 2). Furthermore, the endothelial factors’ levels observed between the lower tertile and the upper tertile exhibited an approximately two-fold difference in the PPL group (Table 2). The similar increases in inflammation factors such as endocan and other endothelial factors are observed in the PPL group. These inflammatory factors appear to be at the highest levels in the third tertile of the PPL group. 

Pawlak et al. noted a significant relationship between endocan levels and adhesion molecules, such as sICAM-1 and sVCAM-1, and inflammation molecules such as TNF-a, thus emphasizing the effect of endocan on vascular and inflammatory mechanisms [36]. Tadzic et al. confirmed the positive correlation of endocan with ICAM-1 in hypertensive subjects [37]. In addition, Bar et al. determined the positive associated of endocan with IL-6 proinflammatory cytokine [35]. One of the intriguing findings of the present study is that the AUC exhibited a positive significant correlation with other endothelial and inflammation factors levels (Table 3, Figure 2 and Figure 3). Taken together, our findings indicate that with the increase in endocan levels in subjects with PPL, there is an activation of the endothelial cells, followed by changes and increases in circulating adhesion (ICAM-1, VCAM-1, VEGFA) and inflammatory (IL-6 and LFA-1α) molecules. This in turn may indicate that the accompanying increased endocan and levels of other endothelial and inflammation factors in the PPL group may be considered one of the important risk factors for the development of atherosclerosis.

ROC analysis was applied to determine the status of endocan level in terms of endothelial dysfunction markers and to determine its place among these markers in PPL. Serum endocan level showed a significant AUC value (AUC = 0.996, *p* = 0.0001, Figure 3). Endocan level therefore performed similarly to established endothelial factors sVCAM-1, sICAM-1, and VEGFA for the PPL group.

The limitation of the current study is the relatively small sample size, but it is also suitable for the number of samples obtained as a result of the G power test. The relationship between AUC and endocan levels in the PPL group could be significantly demonstrated by increasing the number of individuals, particularly in the tertiles. More comprehensive studies are needed in line with the results of the current study. In particular, future research should address the mechanisms involved in the high postprandial endothelial activation and focus on its relationship with atherosclerosis in individuals with PPL.

## 5. Conclusions

It was concluded that circulating endocan levels are significantly increased and independently associated with endothelial and inflammatory factors in PPL. It also showed a close association with the degree of PPL. One could speculate that increased levels of endocan in PPL may promote the development of endothelial cell dysfunction. Additionally, circulating endocan levels were determined to be useful and reliable in the assessment of endothelial dysfunction in PPL and dyslipidemia. 

## Figures and Tables

**Table 1:** Anthropometric measurements and biochemical variables in the study group.

Parametreler	Control *n*:28	PPL *n*:54	*p*
Age (years)	42.0 ± 6.76	45.6 ± 8.42	0.786
BMI (kg/m^2^)	23.8 ± 2.68	27.3 ± 4.75	0.0001
WHR	0.802 ± 0.071	0.865 ± 0.096	0.022
WHtR	0.477 ± 0.052	0.549 ± 0.078	0.0001
Glucose (mmol/L)	4.96 ± 0.361	5.30 ± 0.512	0.012
Glucose TG 4th h (mmol/L)	5.07 ± 0.373	5.76 ± 0.546	0.376
Insulin (pmol/L)	38.7 (21.7–67.1)	45.0 (30.7–54.0)	0.286 *
Insulin TG 4th h (pmol/L)	43.3 (28.6–49.3)	49.6 (34.3–58.5)	0.122 *
HOMA-IR	1.44 (0.821–2.76)	2.08 (1.22–2.83)	0.129 *
TG fasting (mmol/L)	0.965 (0.711–1.16)	2.20 (1.55–2.89)	0.0001 *
Postprandial TG 4th h (mmol/L)	1.46 (1.20–1.74)	2.94 (2.09–3.74)	0.0001 *
TC (mmol/L)	4.65 ± 0.701	5.50 ± 0.938	0.0001
HDL-C (mmol/L)	1.51 ± 0.380	1.25 ± 0.311	0.005
LDL-C (mmol/L)	2.80 ± 0.451	3.25 ± 0.635	0.0001
RLP-C (mmol/L)	0.518 (0.206–0.725)	0.958 (0.458–1.79)	0.002 *
AIP (mmol/L)	0.176 (−0.026–0.255)	0.583 (0.420–0.769)	0.0001
AUC of fasting and 4th h TG	428 ± 116	920 ± 230	0.0001

The *p*-value shows differences between control and PPL groups according to Student’s *t*-test. Data were expressed as mean ± SD. * The *p*-value shows differences between control and PPL groups according to Mann–Whitney U test. Data were expressed as median (interquartile range for 25–75%). BMI: body mass index, WHR: waist-to-hip ratio, WHtR: waist-to-height ratio, HOMA-IR: homeostatic model assessment for insulin resistance, TG: triglyceride, TC: total cholesterol, HDL-C: high-density lipoprotein cholesterol, LDL-C: low-density lipoprotein cholesterol, RLP-C: remnant lipoprotein cholesterol, AIP: atherogenic index of plasma, AUC: area under the curve, *p* < 0.05.

**Table 2:** Fasting serum levels of endothelial and inflammatory factors in control and PPL tertiles according to AUC, mean ± SD.

				AUC of Fasting and 4 h TG Tertiles			
	Control 426 ± 116 (*n*:28)	PPL 920 ± 230 (*n*:54)	*p*	1 539 ± 158 (*n*:18)	2 958 ± 93 (*n*:18)	3 1284 ± 114 (*n*:18)	*p*
Endothelial factors							
Endocan, pg/mL	43.1 (32.3–52.8)	145 (107–188)	0.0001	101 (83.0–118) ^a^	140 (120–162) ^a,b^	201 (177–281) ^a,b,c^	0.0001 *
VEGFA, pg/mL	37.2 (33.0–53.1)	141 (93.6–190)	0.0001	79.9 (71.4–93.8) ^a^	168 (136–194) ^a,b^	189 (145–228) ^a,b^	0.0001 *
sICAM-1, pg/mL	135 (110–181)	529 (301–960)	0.0001	236 (189–301) ^a^	562 (447–799) ^a,b^	1176 (841–1259) ^a,b, c^	0.0001 *
sVCAM-1, ng/mL	8.77 (5.97–8.72)	22.1 (14.3–31.3)	0.0001	12.0 (10.59–15.6) ^a^	22.1 (16.4–25.2) ^a,b^	36.9 (31.2–46.7) ^a,b,c^	0.0001 *
Inflammatory factors							
IL-6, pg/mL	11.3 (10.0–14.5)	24.4 (15.7–25.7)	0.0001	15.0 (12.0–15.8) ^a^	24.8 (23.7–25.9) ^a,b^	25.5 (25.1–31.8) ^a,b^	0.001 *
LFA-1α, ng/mL	0.236 (0.230–0.243)	0.326 (0.281–0.388)	0.0001	0.247 (0.242–0.283) ^a^	0.342 (0.316–0.388) ^a,b^	0.384 (0.336–0.401) ^a,b^	0.001 *

The *p*-values according to Mann–Whitney U test. * The *p*-values according to Kruskal–Wallis test, post hoc Mann–Whitney U test. Data were expressed as median (interquartile range for 25–75%). ^a^ significantly different from control, ^b^ significantly different from tertile 1, ^c^ significantly different from tertile 2, *p* < 0.05. AUC: area under the curve, endocan: endothelial-cell-specific molecule-1, VEGFA: vascular endothelial growth factor A, sICAM-1: soluble intercellular adhesion molecule-1, sVCAM-1: soluble vascular cell adhesion molecule-1, IL-6: interleukin-6, LFA-1α: lymphocyte-function-associated antigen-1 alpha.

**Table 3:** Correlation coefficients of endothelial, inflammatory, and atherogenic factors with AUC and Endocan in PPL group.

PPL (*n*:54)				
	AUC of Fasting and 4 h TG		Endocan	
	Spearman’s Rho	*p*	Spearman’s Rho	*p*
Endothelial factors				
Endocan, pg/mL	0.768	0.003		
sICAM-1, pg/mL	0.856	0.001	0.694	0.007
sVCAM-1, ng/mL	0.883	0.001	0.723	0.001
VEGFA, pg/mL	0.622	0.008	0.709	0.003
Inflammatory factors				
IL-6, pg/mL	0.652	0.006	0.658	0.005
LFA-1α, ng/mL	0.735	0.004	0.587	0.010
Atherogenic factors				
RLP-C	0.465	0.037	0.503	0.031
AIP	0.757	0.001	0.817	0.001

The *p*-value according to Spearman tests. AUC: area under the curve, endocan: endothelial-cell-specific molecule-1, VEGFA: vascular endothelial growth factor A, sICAM-1: soluble intercellular adhesion molecule-1, sVCAM-1: soluble vascular cell adhesion molecule-1, IL-6: interleukin-6, LFA-1α: lymphocyte-function-associated antigen-1 alpha, RLP-C: remnant lipoprotein cholesterol, AIP: atherogenic index of plasma, *p* < 0.05.

## Data Availability

The datasets generated during and/or analyzed during the current study are available from the corresponding author on reasonable request.
